# The effectiveness of aerobic training, cognitive behavioural therapy, and energy conservation management in treating MS-related fatigue: the design of the TREFAMS-ACE programme

**DOI:** 10.1186/1745-6215-14-250

**Published:** 2013-08-12

**Authors:** Heleen Beckerman, Lyan JM Blikman, Martin Heine, Arjan Malekzadeh, Charlotte E Teunissen, Johannes BJ Bussmann, Gert Kwakkel, Jetty van Meeteren, Vincent de Groot

**Affiliations:** 1 Department of Rehabilitation Medicine, VU University Medical Center, PO Box 7057, Amsterdam, MB 1007, The Netherlands; 2 EMGO Institute for Health and Care Research, VU University Medical Center, PO Box 7057, Amsterdam, MB 1007, The Netherlands; 3 MS Center Amsterdam, PO Box 7057, Amsterdam, MB 1007, The Netherlands; 4 Department of Rehabilitation Medicine and Physical Therapy, Erasmus MC-University Medical Center, PO Box 2040, Rotterdam, CA 3000, The Netherlands; 5 Rudolf Magnus Institute of Neuroscience and Center of Excellence for Rehabilitation Medicine, University Medical Center Utrecht and Rehabilitation Center, De Hoogstraat, Rembrandtkade 10, Utrecht, TM 3583, The Netherlands; 6 Department of Clinical Chemistry, VU University Medical Center, PO Box 7057, Amsterdam, MB 1007, The Netherlands

**Keywords:** Multiple sclerosis, Fatigue, Aerobic training, Energy conservation management, Cognitive behavioural therapy, Biomarkers, HPA-axis, Cortisol, Cytokines, Rehabilitation medicine, Randomised controlled trial

## Abstract

**Background:**

TREFAMS is an acronym for TReating FAtigue in Multiple Sclerosis, while ACE refers to the rehabilitation treatment methods under study, that is, Aerobic training, Cognitive behavioural therapy, and Energy conservation management. The TREFAMS-ACE research programme consists of four studies and has two main objectives: (1) to assess the effectiveness of three different rehabilitation treatment strategies in reducing fatigue and improving societal participation in patients with MS; and (2) to study the neurobiological mechanisms of action that underlie treatment effects and MS-related fatigue in general.

**Methods/Design:**

Ambulatory patients (*n* = 270) suffering from MS-related fatigue will be recruited to three single-blinded randomised clinical trials (RCTs). In each RCT, 90 patients will be randomly allocated to the trial-specific intervention or to a low-intensity intervention that is the same for all RCTs. This low-intensity intervention consists of three individual consultations with a specialised MS-nurse. The trial-specific interventions are Aerobic Training, Cognitive Behavioural Therapy, and Energy Conservation Management. These interventions consist of 12 individual therapist-supervised sessions with additional intervention-specific home exercises. The therapy period lasts 16 weeks. All RCTs have the same design and the same primary outcome measures: fatigue - measured with the Checklist Individual Strength, and participation - measured with the Impact on Participation and Autonomy questionnaire. Outcomes will be assessed 1 week prior to, and at 0, 8, 16, 26 and 52 weeks after randomisation. The assessors will be blinded to allocation. Pro- and anti-inflammatory cytokines in serum, salivary cortisol, physical fitness, physical activity, coping, self-efficacy, illness cognitions and other determinants will be longitudinally measured in order to study the neurobiological mechanisms of action.

**Discussion:**

The TREFAMS-ACE programme is unique in its aim to assess the effectiveness of three rehabilitation treatments. The programme will provide important insights regarding the most effective treatment for MS-related fatigue and the mechanisms that underlie treatment response. A major strength of the programme is that the design involves three almost identical RCTs, enabling a close comparison of the treatment strategies and a strong overall meta-analysis. The results will also support clinical practice guidelines for the treatment of MS-related fatigue.

**Trial registrations:**

Current Controlled Trials ISRCTN69520623, ISRCTN58583714, and ISRCTN82353628

## Background

Multiple sclerosis (MS) is a neurodegenerative disease characterised by demyelinisation, axonal loss and inflammation of the central nervous system (CNS). Although the first description of MS dates back to the mid-19th century, in spite of this long history the aetiology is still unknown and no curative treatment is available [1]. The disease is most probably caused by an interplay between immunological, environmental and genetic factors [2,3]. MS affects young and middle-aged people, with women twice as likely to be affected as men, and is known to cause a variety of clinical symptoms such as neurological impairments, fatigue, depression and pain [2].

Fatigue is one of the most often reported and disabling symptoms in MS and restricts societal participation and performance in daily life at home, at work and in leisure activities [4-6]. Although the importance of fatigue as a disabling symptom of MS is widely acknowledged, there is no consensus on the definition of fatigue. DeLuca [7] defines fatigue as the reduction in performance with either prolonged or unusual exertion. Furthermore, fatigue can be sensory, motor, cognitive or subjective. The Multiple Sclerosis Council for Clinical Practice Guidelines [8] defines fatigue in MS as a subjective lack of physical and/or mental energy that is perceived by the individual (or caregiver) to interfere with usual and desired activities. Chaudhuri and Behan [9] distinguish central fatigue from peripheral fatigue, and define central fatigue as the failure to initiate and/or sustain attentional tasks (mental or cognitive fatigue) and physical activities (physical fatigue). Peripheral fatigue is described as muscle fatigability due to disorders of muscle and neuromuscular junctions. The definitions of DeLuca [7], the MS Council for Clinical Practice Guidelines [8] and the concept of central fatigue outlined by Chaudhuri and Behan [9] concur with our view that MS-related fatigue is a multifaceted symptom. Fatigue in MS can also be subdivided into primary and secondary. Primary fatigue relates to specific pathophysiological mechanisms that are the direct consequence of the MS disease process. On the other hand, a number of factors, while not considered primary causes of MS-related fatigue, may be secondary contributors. These factors are not unique to MS, but are the result of symptoms of MS such as sleep problems due to spasm or urinary problems, depression or physical deconditioning. Fatigue might also be a side effect of disease modifying drugs.

Although the exact pathophysiological mechanism behind MS-related fatigue is unknown, it is most likely multifactorial. A number of pathophysiological mechanisms have been proposed including dysregulation of the immune system, dysfunction of the CNS, impaired nerve conduction, neuro-endocrine/neurotransmitter dysregulation, the involvement of the autonomic nervous system and energy depletion [10,11]. Available information can be combined in a biological model in which environmental stressors such as infections, immunisation, trauma and life events influence genetically predisposed variables such as the sensitivity of the hypothalamic-pituitary-adrenal axis (HPA-axis), glucocorticoid receptors and the noradrenaline system [9,12,13]. Consequently, fatigue is triggered in susceptible individuals. In addition to disease-related, genetic and environmental factors, psychological mechanisms may play an important role in causing and sustaining MS-related fatigue [12-14].

In clinical practice, MS-related fatigue is often treated with a combination of therapies, which makes it difficult to distinguish the effect of each therapy component. Due to the limitations of available evidence, current pharmacological approaches to treating MS-related fatigue are mainly based on preliminary studies and expert consensus. Amantadine, Modafinil and Aminopyridine are pharmacological strategies mainly used by neurologists [15]. Current evidence supporting the effectiveness of non-pharmacological interventions such as Aerobic Training (AT) [16-22], Cognitive Behavioural Therapy (CBT) [23] and Energy Conservation Management (ECM) [24-26] on MS-related fatigue is encouraging, but findings are heterogeneous and only a few studies have evaluated MS-related fatigue as the primary outcome measure [27]. Moreover, the methodological quality of non-pharmacological trials is often hampered by issues such as the complexity of the (multidisciplinary) treatment, the lack of adequate control groups, treatment blinding of patients and assessors, and the expertise of the involved therapists. These issues have resulted in an extension of the CONSORT statement for non-pharmacological trials [28]. Systematic reviews of exercise therapy and energy conservation management trials are underway or recently published [29,30].

TREFAMS is an acronym for the TReating FAtigue in MS programme, and ACE refers to the rehabilitation treatment methods under study, that is, Aerobic training, Cognitive behavioural therapy, and Energy conservation management. The programme has two main objectives: (1) to assess the effectiveness of three different rehabilitation treatment strategies in reducing fatigue and in improving societal participation in individual MS patients; and (2) to study the biological mechanisms that underlie treatment effects and MS-related fatigue in general. The TREFAMS-ACE research programme includes three randomised clinical trials (RCTs), and one explanatory study on the biological mechanisms of action that underlie treatment effects and MS-related fatigue in general.

A significant body of evidence now implicates both HPA-axis abnormalities and immune markers in the pathophysiology of MS-related fatigue. MS patients with fatigue exhibited a higher activity of the HPA-axis than patients without fatigue [13,31]. Earlier studies have examined a possible relationship between cytokines and fatigue in MS [32,33]. A study of pro-inflammatory (IFN-γ, TNF-α) and anti-inflammatory (IL-10) cytokine production in MS patients showed that patients with fatigue had a significantly higher production of IFN-γ and TNF-α than patients without fatigue. IL-10 production did not significantly differ between the two groups [32]. Flachenecker and Bihler [33] found a relationship between TNF-α mRNA expression and fatigue, but not between fatigue and IFN-γ or IL-10. In view of a relationship to inflammatory markers found in two independent studies and the higher HPA-axis reactivity in MS, we hypothesise that fatigue in patients with MS is stress-related and that it is caused by an inflammatory mechanism. In addition, we assume that the extent of imbalance between pro-inflammatory and anti-inflammatory cytokines is associated with the severity of fatigue.

AT is aimed at improving physical fitness and at reducing an inactive, deconditioning lifestyle. Improved physical fitness may lead to normalisation of HPA-axis functioning [34], a reduction in pro-inflammatory cytokines and/or an increase in anti-inflammatory cytokines [35], leading to a reduction in MS-related fatigue. We hypothesise that an improved physical fitness due to AT will be accompanied by reduced fatigue and, as a consequence, improved societal participation.

CBT focuses on fatigue-maintaining cognitions and behaviour, examples of which are insufficient coping with MS or MS-related fatigue, fear of disease progression, dysregulation of activity or sleep, low social support and focusing on fatigue. The general aim of CBT is to improve daily functioning and to decrease fatigue by changing fatigue-maintaining cognitions and behaviour, within the limits of the MS [36]. We hypothesise that CBT may reduce perceived stressors (for example, environmental, psychological and biological), and consequently may lead to normalisation of HPA-axis functioning and cytokine profiles.

ECM includes energy conservation strategies, ergonomic advice and coaching aimed at more efficient use of available energy. Energy conservation strategies have been defined as the identification and development of activity modifications to reduce fatigue through a systematic analysis of daily work, home and leisure activities in all relevant environments [8]. Packer et al. [37] were the first to develop an ECM treatment protocol for a 6-week group course. In a clinical trial, this group course proved to be effective in patients with MS, both immediately following the course and after 1 year [24,25]. The treatment goal of ECM is to promote a positive attitude aimed at stimulating active decision-making and the optimal use of available energy in relation to the unique needs of each individual. We hypothesise that ECM may lead to a reduction in environmental and psychological stressors and consequently to the normalisation of biological stressors (HPA-axis functioning, cytokines), which may in turn lead to reduced fatigue and improved participation.

Accordingly, the following research questions have been formulated in the TREFAMS-ACE programme:

1. What is the effectiveness of Aerobic Training on fatigue and participation? Can this effect be attributed to an increase in fitness parameters?

2. What is the effectiveness of CBT on participation and fatigue? Can this effect be explained by altered cognitions regarding fatigue?

3. What is the effectiveness of Energy Conservation Management advice on fatigue and participation? Can this effect be attributed to the implementation of ergonomic advice or adherence to altered time-schedules?

4. Which treatment strategy reduces fatigue and improves participation most effectively?

5. Does effective therapy lead to normalisation of HPA-axis function, a reduction in pro-inflammatory cytokines or an increase in anti-inflammatory cytokines?

## Methods/Design

### Design

**Figure 1:**
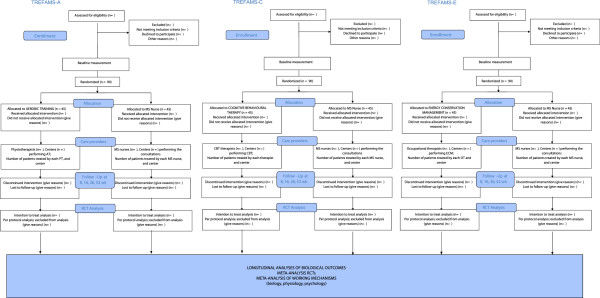
The design of the TREFAMS-ACE programme.

TREFAMS-ACE is a multicentre programme that includes three single-blinded RCTs with repeated measurements in time, in which the effectiveness of Aerobic Training, CBT and Energy Conservation Management on MS-related fatigue and participation in patients with MS will be investigated. All RCTs will use the same two-parallel-arms design (Figure 1), the only difference being the specific intervention applied [28]. Patients will be randomised to receive either a high-intensity trial-specific treatment, which consists of a series of 12 therapist-led sessions in 4 months, or a low-intensity treatment by an experienced MS-nurse, which consists of three consultations in 4 months. Participants will be followed for 1 year.

In addition to the three clinical trials, a fourth study has been defined that will focus on biological outcome measurement and understanding the biological mechanisms of action underlying MS-related fatigue [38,39]. This study should also help to improve our understanding of the biological mechanisms of the four interventions under study (see Figure 1).

The medical ethics committee of the VU University Medical Center approved the TREFAMS-ACE programme. Additionally, local feasibility statements were obtained from each participating medical centre.

### Participants

The 270 adult patients (90 patients per RCT, and 45 per intervention group) required will have to fulfill the following inclusion criteria:(a) definitive diagnosis of MS; (b) severely fatigued; (c) ambulatory patients; (d) no evident signs of an exacerbation, or a corticosteroid treatment in the past 3 months; (e) no current infections; (f:) no anaemia; (g) a normal thyroid function. The exclusion criteria are: (a) depression; (b) primary sleep disorders; (c) severe co-morbidity; (d) current pregnancy or having given birth in the past 3 months; (e) pharmacological treatment for fatigue that was started in the past 3 months (for example, Amantadine, Modafinil, Ritalin, Pemoline); (f) non-pharmacological therapies for fatigue that took place in the past 3 months. See Table 1 for the operationalisation of the inclusion and exclusion criteria.

**Table 1:** Inclusion and exclusion criteria TREFAMS-ACE trials

**Inclusion criteria**	**Exclusion criteria**
Definitive diagnosis of MS	Depression (HADS depression >11)
Severely fatigued (CIS20r-fatigue ≥35)	Primary sleep disorders
Aged between 18 and 70 years	Severe co-morbidity (CIRS item scores ≥3)
Ambulatory patients (an EDSS score ≤6)	Current pregnancy or having given birth in the past 3 months
No evident signs of an exacerbation or a corticosteroid treatment in the past 3 months	Pharmacological treatment for fatigue in the past 3 months
No infections (normal leukocytes and C-reactive protein in blood)	Non-pharmacological therapies for fatigue in the past 3 months
No anaemia (normal haemoglobin and haematocrit in blood)	
No thyroid dysfunction (normal thyroid stimulating hormone (TSH) in blood)	

### Recruitment strategy

To avoid contamination of interventions, each RCT will be carried out at a different (university) medical centre: the AT study will be conducted in the St Antonius Hospital, Nieuwegein, in collaboration with the University Medical Center Utrecht, the CBT study will be conducted at the VU University Medical Center in Amsterdam and at the University Medical Centre Nijmegen (UMCN), and the ECM study will be conducted at the Erasmus MC-University Medical Center, Rotterdam, and Rehabilitation Center Leijpark in Tilburg. Patients will initially be recruited through the participating main study centres. The Dutch patient organisation Multiple Sclerosis Vereniging Nederland (MSVN) has been involved since the design phase of the research programme, and has offered to help with recruitment. If the main study centres are not able to recruit sufficient participants, recruitment will also take place in hospitals and rehabilitation centres in the neighbourhood of the main study centres. MS-nurses, neurologists and residents in neurology and rehabilitation medicine will inform potentially eligible patients about the TREFAMS-ACE study. A neurologist or a rehabilitation physician will check the inclusion and exclusion criteria listed in Table 1 in the case of a potentially eligible patient. Subsequently, patients eligible for participation will be asked to complete an informed consent form before participating in the study.

### Interventions

#### Aerobic training (AT)

AT aims to improve the participant’s fitness and consists of 12 physiotherapist-led exercise sessions on a bicycle ergometer (Table 2). Moreover, participants will be provided with an identical bicycle ergometer at home on which they will be asked to perform additional training sessions, leading to the recommended three sessions per week. Each 30-min interval-type training session consists of six cycles of 5 min. Each cycle consists of 3 min of low intensity exercise, 1 min of moderate intensity exercise and 1 min of high intensity exercise. At the start of the treatment and after 8 weeks of training, current fitness levels will be assessed with a graded maximal exercise test to volitional exhaustion. Following the 8-week maximal exercise test, exercise intensities will be adjusted to meet the newly obtained fitness level. After completion of the supervised training programme, patients will be encouraged to continue exercising and to remain physically active. Generally, physiological adaptations to aerobic training occur when: (1) the training intensity is at least 60% VO2max, and (2) the training is carried out at least three times a week [40]. The current treatment protocol addresses both factors.

**Table 2:** AT programme

**45-min session of bicycle ergometer training**	**Details**
Warming-up	• 5 min at 25% to 40% Wmax
Aerobic training	• Six cycles of 5 min: 3 min 40% Wmax, 1 min 60% Wmax and 1 min 80% Wmax
	• Cadence: 60-80 revolutions per min (rpm)
	• Heart rate should not exceed 80% of the predicted maximal heart rate
	• Training intensity will be updated once during the training, according to the 8-week maximal exercise test
	• The work rate can be adjusted, based on the clinical expertise of the supervising physiotherapist
Cooling down	10 min
	• All training sessions and adjustments to the work rate are recorded in the training log
Home exercises	Participants will be provided with an identical bicycle ergometer at home so that they can perform additional training sessions, leading to the recommended three sessions per week

#### Cognitive behavioural therapy (CBT)

CBT is directed at behaviours or cognitions that perpetuate fatigue. Examples are dysfunctional cognitions with respect to MS, fatigue or pain, persistent focusing on symptoms, deregulation of physical and social activities and a lack of social support [41,42]. It is thought that fatigue will decrease if these perpetuating factors are identified by the patient him/herself and changed. CBT consists of 12 sessions over a 4-month period. Ten different modules have been developed to target specific fatigue maintaining factors [43] (Table 3). CBT will be customised to each individual patient using indicator criteria for each module that are based on cutoff scores on questionnaires and on a diagnostic interview (Table 3). In the final therapy sessions, special attention will be paid to integrating the skills obtained into daily life and how to handle behavioural relapses. The effectiveness of this theory-based CBT strategy has already been investigated in several other patient populations and healthcare settings [44-48].

**Table 3:** CBT modules

**Module**	**Questionnaires and instruments**
1. Formulating goals	For all participants
This module applies to all participants. Concrete and obtainable treatment goals are formulated during therapy. Goals comprise activities that the participant wishes to do when the fatigue has decreased or disappeared.	
2. Sleep/wake rhythm	SIP sleep and rest ≥60 [49]
The importance of a regular sleep/wake rhythm and good sleep hygiene is explained to the patient. Furthermore, the sleep/wake rhythm of will be discussed and suggestions for improvement given.	
3. Beliefs regarding MS	Impact of Event Scale (IES) ≥20 [50]
Participants will receive realistic information about MS. Dysfunctional cognitions about MS or the future are identified and challenged, and the participant is supported in forming more functional cognitions. Problems regarding acceptance of the disease are also addressed.	Pictorial Representation of Illness Measure (PRISM): Burden of MS heavier than burden of fatigue [51]
	Illness Cognition Questionnaire (ICQ), concentration ≤12 [52]
	Cognitive Behavioural Responses to Symptoms Questionnaire (CBRSQ) [53,54]:
	Resting behaviour >14,3;
	All-or-nothing behaviour >12.9;
	Symptom focusing >15.5;
	Catastrophising >12.6;
	Embarrassment >16.4;
	Damage >20.5;
	Fear avoidance >15.3
	HADS [55]
	Depression >9
	Anxiety >9
	Fear of disease Progression Questionnaire (FoP-Q), ≥4 on at least 75% of the 34
	Anxiety items [56,57]
4. Beliefs regarding fatigue	SES-28 fatigue ≤19
Participants are supported in changing dysfunctional views about fatigue such as a lack of self-efficacy, catastrophising fatigue and somatic attributions.	Jacobsen Fatigue Catastrophising Scale ≥16 [58,59]
5. Focusing on fatigue	Illness Management Questionnaire (IMQ), focusing on symptoms ≥4 [60]
The concept of persistent focusing on fatigue and its consequences are discussed. Participants practise redirecting their attention from fatigue to activities and other sensations. Talking about fatigue is discouraged.	
6. Regulation of physical activity	Activity Interview and Activity Monitor
Depending on their level of activity, participants learn how to divide their activities, followed by a systematic increase in regular physical activity to obtain predefined goals.	
7. Regulation of social activity	SIP social interaction ≥100 [49]
Patients are empowered to expand social activities and deal with problems that can arise during social interaction.	SF36 social functioning ≤65 [61]
8. Regulation of mental activity	CIS20r concentration ≥18 [62]
Participants are supported with regards to practising and expanding mental activities such as working on the computer or reading. Participants learn how to deal with possible cognitive deficits such as concentration or memory problems.	
9. Role of the environment	Social Support List (SSL) [63]
Unrealistic expectations of the environment are addressed and more realistic expectations are promoted. Participants learn how to express their limits and boundaries to ‘significant others’.	Discrepancies ≥50;
	Negative interactions ≥14
10. Handling pain	SF36 bodily pain ≤60 [61]
Dysfunctional cognitions about pain are challenged and replaced by more functional cognitions.	Pain Catastrophising Scale (PCS) ≥16 [64]

#### Energy conservation management (ECM)

The ECM treatment protocol is based on a group course for energy conservation developed by Packer et al. [37]. The TREFAMS intervention, called Individual ECM treatment (IECM), is individualised and consists of 12 45-min sessions over 4 months, given by a trained occupational therapist. For the IECM, the original content of the Packer et al. group programme will be divided up to fit the 4-month treatment period. Attention will also be paid to individual learning and approaching styles to assimilate the programme contents. Motivational interviewing will be used as a communication technique to assist patients in exploring and resolving ambivalence to change. Table 4 shows the content of the IECM. A variety of teaching methods will be used, including providing information, discussions, long-term and short-term goal setting, practice activities and homework activities, to assist the patient’s integration of energy conservation principles into the performance of everyday tasks. The aim of ECM is not so much to correct the underlying mechanisms of fatigue, nor to accept that the solution is to decrease activity levels or reduce the breadth and extent of activities. Instead, the aim is to promote a positive attitude aimed at active decision-making and the optimum use of the available energy to fit the unique needs of each individual. ECM is also intended to reduce the impact and severity of fatigue, to increase patients’ use of energy-conserving strategies and to improve their confidence in their ability to manage fatigue [37].

**Table 4:** Individual energy conservation management

**Sessions**	**Content of the sessions**
Introduction session	• Getting to know the patient, identification of problems in daily life with help of the COPM, impact of fatigue on daily life
	• Hand out workbook IECM, activity list per day/week to give insight in load and loadability of the patient, and learning style assessment
	
Analysis of the problems	• Discuss activity/participation problems, outcomes of load and loadability from the activity lists
	• Analysis of problems, determine questions of help, and the learning and approaching style
	• Formulate the problems and treatment goals
	
Treatment sessions	a. Information about fatigue
	• types, causes and factors influencing fatigue
	• banking (saving) and budgeting (deciding how to spend) energy
	b. Importance of rest
	• how fatigue can influence your daily life
	• rest as a way of relieving fatigue
	c. Balancing your schedule
	• components of a balanced lifestyle
	• how to balance (light and heavy) activities
	• planning a weekly schedule
	d. Communication
	• expressing needs to others
	• breaking down negative attitudes about fatigue and rest
	e. Priorities and standards
	• breaking down activities in order to simplify them as much as possible
	• budgeting energy, making decisions about priorities and standards
	f. How to do activities
	g. Ergonomics, body positions and assistive devices
	• organisation of needed environments (work, home) to promote good body mechanics
	• Organisation of needed environments to save energy
	• Technology and equipment that can save energy
	• Structure of body/biomechanics
	• How to use body properly/ergonomics
Evaluation session	

#### MS-nurse consultations

The low-intensity treatment by experienced MS-nurses consists of three consultations of 45 min over a 4-month period. The content of the consultations led by the MS-nurse will cover two important aspects to control for: (1) reliable information on MS-related fatigue; and (2) attention from an experienced MS professional in order to reassure the patient that his/her concerns or questions will be taken seriously [65]. In the first consultation, the patient receives a booklet containing general information about MS-related fatigue and factors that may influence fatigue. This booklet was designed by the TREFAMS-ACE research team to provide patients with standardised information about fatigue, without adding details regarding specific interventions so as to avoid overlap with the trial-specific interventions. In the remaining two consultations, participants will have the opportunity to discuss their personal experiences in coping with fatigue, ask questions about the booklet and discuss other fatigue-related issues. The consultations with the MS-nurses should not be considered as ‘usual care’ because, due to the TREFAMS-ACE study design, the MS-nurses are restricted in referring patients to a psychologist, physiotherapist or other healthcare professional within the hospital. In the Netherlands, timely referral is an important aspect of normal MS-nursing practice.

#### Therapist training

All involved therapists were selected based on their experience with the intervention and with treating MS patients. Furthermore, all received training that was focused on one of the four treatment protocols used in the TREFAMS-ACE study.

Physiotherapists experienced in cardio-respiratory training were introduced to the exercise protocol, the use of the bicycle ergometer, study materials and measurements in TREFAMS-AT.

The psychologists involved in the TREFAMS-CBT study all received an additional 4-day CBT training course at the Expert Centre Chronic Fatigue of UMCN. The training course consisted of an introduction to the protocol, training in the content of each treatment module and how to determine which modules are indicated for a specific participant. The skills needed to change patient cognition and behaviours were practised during role-playing, with the help of simulated patients. To ensure the quality of the CBT, weekly peer conversations between therapists, in which experiences with the TREFAMS-CBT participants will be shared, are part of the therapist training.

Occupational therapists already familiar with energy conservation strategies and the Packer group course ‘Managing Fatigue’ received a 1-day training course in the implementation of the individual Energy Management Course (IECM). This course was given by the researcher, together with an expert therapist in energy conservation management. Training consisted of a thorough explanation of the content of the 12 sessions and how sessions can be individually tailored. In addition, occupational therapists not yet qualified in applying Motivational Interviewing had to attend a 3-day Motivational Interviewing course.

All MS-nurses involved in one of the RCTs participated in a 1-day training course. In this course the MS-nurses shared their approach to taking a fatigue-related nursing history, they were instructed as to how to provide relevant information on MS-related fatigue without giving concrete therapeutic advice, and they were informed of the restrictions concerning the referral of patients to other healthcare professionals within the hospital (MS team members). These newly-learned skills were practised using role-playing.

### Outcome measures

Outcome measures consist of validated self-reported questionnaires, blood and saliva, activity monitoring and physical fitness tests. All primary and secondary outcomes will be assessed 1 week prior to, and at 0, 8, 16, 26 and 52 weeks after randomisation. The self-reported questionnaires will be offered to patients via the internet or on paper, and will be completed at home. Within each self-reported questionnaire, the sequence of questions will be randomised between measurement occasions. The drawing of blood samples (according to the study protocol), physical fitness tests and assessor-based interviews will take place at the outpatient clinic of the participating centres. Saliva sampling will take place at home and includes several time-points per day.

#### Primary outcome measures

1. Fatigue will be measured with the Checklist Individual Strength (CIS20r), domain fatigue [62,66]. This multidimensional questionnaire consists of 20 items, divided into four dimensions of fatigue and related behavioural aspects, including: (a) the subjective experience of fatigue (8 items); (b) reduction in motivation (4 items); (c) reduction of physical activity (3 items); and (d) reduction in concentration (5 items). The CIS20r focuses on fatigue in the past 2 weeks. Each item is answered using a 7-point scale. The CIS20r fatigue score is a sum score that can vary between 8 and 56 points. Recently, the reproducibility, distribution-based responsiveness and concurrent validity of the CIS20r were investigated in patients with MS [67]. Despite good test-retest reliability, a smallest detectable change of 11.8 points was found, leading to the recommendation to monitor trial participants repeatedly over time using a set of complementary fatigue scales [67]. A systematic review of the measurement properties of 31 fatigue questionnaires confirms this recommendation [68].

2. Societal participation will be assessed with the Impact on Participation and Autonomy questionnaire (IPA) [69]. The IPA questionnaire was developed to assess the severity of restrictions in participation and individual needs related to participation and autonomy. The IPA is a generic questionnaire that addresses: (a) perceived participation, reflected in 31 items in five domains, that is, autonomy indoors, autonomy outdoors, family role, social relations, work and education; and (b) the experience of problems related to every aspect of participation, reflected in eight problem experience scores [69]. An anchor-based responsiveness study in a heterogeneous outpatient rehabilitation population showed that the IPA was moderately able to detect within-patient improvement over time [69]. No studies on the responsiveness and minimal important change of the IPA in patients with MS are yet available [70].

#### Secondary outcome measures

MS-related fatigue is a multifaceted symptom with various types of expression. Therefore, several other fatigue measures are also included. The impact of fatigue will be measured with the Modified Fatigue Impact Scale (MFIS) and the Fatigue Severity Scale (FSS) [71,72]. The MFIS assesses the effects of fatigue in terms of physical, cognitive and psychosocial functioning. The FSS evaluates the severity and impact of fatigue in patients with MS. A patient-reported diurnal course of fatigue during 1 day will be assessed using short message services (SMS) technology. Moreover, the Rehabilitation Activities Profile (RAP) and the Medical Outcome Study Short Form 36 (SF36) will be used to measure daily functioning and participation [61,73].

### Determinants

These include descriptive variables, mediators (that is, intervening causal variables), confounding or effect-modifying factors that have been shown to be related to the interventions, and fatigue and participation in MS patients [53,74]. In multifactorial, complex situations such variables can act in different ways in different situations or different analyses and will therefore be further specified in forthcoming articles.

#### Demographic and disease characteristics

Demographic information includes age, gender, ethnicity, living situation, level of education, work and income. The disease-related variables that will be assessed include the type of MS, neurological symptoms, the Expanded Disability Status Scale (EDSS), the number of exacerbations in the year prior to inclusion, the use of disease modifying drugs and other medication, co-morbidities and healthcare use. The EDSS and the Cumulative Illness Rating Scale (CIRS) will be assessed by a physician at baseline and after 52 weeks [75-77]. Cognitive deficits will be assessed at baseline by the Mini Mental State Examination [78].

#### Physical activity and physical fitness

The amount of physical activity and frequency of movement will be registered by means of a tri-axial activity monitor (ActiGraph GT3X+) that will be worn for 7 days. The Physical Activity Scale for Individuals with Physical Disabilities (PASIPD) is a self-reported questionnaire that assesses physical activity over the preceding 7 days. The PASIPD assesses physical activity in three domains: recreation, household and occupation activities [79,80].

In a subgroup of participants, cardio-respiratory fitness will be assessed by means of a maximum capacity test (VO2max test). Participants will perform this test on an electromagnetic bicycle ergometer. Work rate will be progressively increased by 25 + 10 W/min (women) or 25 + 15 W/min (men) until volitional exhaustion, rpm <45 or for safety reasons. In addition, patients will walk as far as possible during a 2-min walk test (2MWT).

#### Cognitive and behavioural factors

Coping style is measured with the Coping Inventory for Stressful Situations (CISS-21) [81,82]. Three main coping styles can be distinguished: task-oriented coping, emotion-oriented coping and avoidance coping. The General Self Efficacy Scale will be used to assess optimistic self-beliefs for coping with a variety of difficult life demands [83]. Possible mood disorders will be assessed with the Hospital Anxiety and Depression Scale (HADS) [55]. The HADS consists of two subscales: depression and anxiety. The tendency to fall asleep during daytime is measured with the Epsworth Sleepiness Scale [84]. Perceptions of fatigue will be measured with the Brief Illness Perception Questionnaire (B-FPQ), which assesses cognitive and emotional representations of fatigue [53]. The B-FPQ is an adaptation of the Brief Illness Perception Questionnaire (B-IPQ), which measures illness perceptions [85]. Fear of progression of MS is measured with the Fear of disease Progression Questionnaire (FoP-Q) [56,57]. Five factors are distinguished: affective reactions, partnership/family, work, loss of autonomy and coping. The Illness Cognitions Questionnaire (ICQ) measures three different generic illness cognitions: helplessness, acceptance and disease benefits [52]. The Social Support List is used to measure the level of social interactions, discrepancies and negative interactions [63].

#### Biological markers

An important part of the TREFAMS research programme concerns the integration and longitudinal study of clinical parameters and biological parameters [38]. HPA-axis functioning will be assessed through the collection and analysis of salivary cortisol. To determine the Cortisol Awakening Response (CAR), saliva will be collected immediately after awakening and after respectively 30 min, 45 min and 60 min post-awakening. Following the fifth sample at 22:00 the participant takes 0.5 mg dexamethasone (low dose dexamethasone suppression test) and saliva is again collected the next morning, immediately after awakening.

Blood will be drawn to determine levels and activity of pro-and anti-inflammatory cytokines [39,86]. Blood and saliva will be collected at the same fixed time-points as for the other outcome measures.

#### Energy saving strategies

To assess which strategies the participants in all three RCTs use to influence their fatigue, we developed the Fatigue Strategies Questionnaire (FSQ). This questionnaire is based on the Energy Conservation Strategies Survey (ECSS) [87] to which we have added a number of strategies on physical activity, cognition and behaviour. Participants will be asked about the strategies they use and how effective these strategies are. To facilitate the meta-analysis of ECM interventions, the participants of the TREFAMS-ECM trial will fill in the original ECSS at 16 weeks [87].

### Sample size

Sample size was calculated based on the CIS20r subscale fatigue. In order to detect a clinically relevant difference of 8 points on the CIS20r subscale fatigue between the study groups in an MS population, with a SD of 12.7, a power of 80%, an alpha of 0.05 and an attrition rate of 20%, 45 patients per group will be needed [47,88]. This amounts to 90 patients for each trial and 270 patients for the entire TREFAMS-ACE programme. Sample size calculation was not adjusted for longitudinal data analyses with repeated measures [89], or for an eventual clustering by care providers or participating centres. Balancing these two factors, we expect that the power of our study will be >80%.

### Randomisation

Patients eligible for participation in the study will be randomised to either the trial-specific intervention or the consultations with the MS-nurse after the baseline measurements have been completed. The randomisation scheme is computer-generated with random variable block sizes. An independent investigator within each main study centre will need to login to the web-based randomisation facility to carry out the randomisation, and will inform the patient and the therapist as to the therapy allocation.

### Blinding

The assessors responsible for physical fitness tests and interviewer-based measures will be told in which week patients need to be measured but will not know which treatment patients receive. Patients will be instructed not to disclose which treatment they are receiving. Furthermore, the analyses of blood and salivary in the clinical chemistry laboratory, and the statistical analyses of the between-group differences, will be performed by research staff blinded to the treatment allocation of the participants.

### Serious adverse events

Based on previous research, AT, CBT, ECM and consulting the MS-nurse are expected to be safe treatment methods in patients with MS [16-21,23-26,90,91]. However, all therapists and assessors involved in the studies will be instructed to report all serious adverse events (SAE) to the principal investigators, after which they will be reported to the Medical Ethical Committee. An SAE is any untoward medical occurrence in a participant that is not necessarily associated with the treatment, but that is lethal, and/or threatens the life of the participant, and/or requires hospitalisation or prolongation of existing hospitalisation, and/or causes persistent or significant disability or incapacity.

### Treatment fidelity and compliance

Data regarding therapy compliance will be subtracted from the administrative hospital databases and therapist notes. Participants will receive an overview of all appointments prior to the first session. If participants cancel or do not attend a session, this session will be rescheduled within the 16-week time frame. Because therapy consists of 12 therapist-led sessions in 16 weeks, some rescheduling is possible. Sessions will not be rescheduled if that means that the 16-week time window will be extended by >1 week.

### Statistical analyses of the RCTs and meta-analyses

The primary analyses of each separate RCT will be based on the intention-to-treat principle using longitudinal data-analysis techniques, such as Generalised Estimating Equations or Hierarchical Linear Mixed Models. To detect the direct effects of the interventions, longitudinal models will be constructed to analyse the differences between the intervention groups regarding the course of within-group changes during the 1-year follow-up period. Furthermore, a meta-analysis of all TREFAMS-ACE data will be conducted to investigate the relative effects of each treatment strategy. In addition, statistical mediation analyses will be used to examine the working mechanisms of the interventions related to, among others, changes in HPA-axis functioning and changes in pro- and anti-inflammatory cytokine levels.

## Discussion

The TREFAMS-ACE study will investigate the effectiveness of three different non-invasive, non-pharmacological rehabilitation treatment strategies aimed at reducing fatigue and improving societal participation in patients with MS. Furthermore, the mechanisms that underlie treatment effects will be studied. This research programme is expected to produce four related PhD-theses. By publication of the design, we wish to be fully transparent as to the quality of the TREFAMS-ACE programme and thus aim to avoid most of the methodological weaknesses reported in current Cochrane reviews in the field of rehabilitation [92-95].

The TREFAMS-ACE study has a number of important strengths, the first of which is a design including three almost identical RCTs on the effectiveness of AT, CBT and ECM, respectively. Our study may provide greater insight into the exact pathophysiological mechanism(s) behind MS-related fatigue and the pathways through which AT, CBT and ECM exert their effect. Because the same design and the same outcome measures are used, at the end of the TREFAMS study an overall analysis can be performed that allows factors to be controlled for that might otherwise cause heterogeneity in a regular meta-analysis of independent RCTs. This will enable a close comparison of the treatment strategies within each trial as well as a strong overall meta-analysis. Second, the large cohort of fatigued MS patients that will be formed will enable us to study the biological mechanisms that explain fatigue and the mechanisms underlying the possible effectiveness of the treatment strategies. Recently, Fischer et al. [38] formulated four criteria for biomarker selection in clinical trials: the biomarker has to be linked to the clinical outcome, that is, MS-related fatigue in our study, and the biomarker should be modifiable in the desired direction. Furthermore, the biomarker should be validly and reliably measured, and finally, the duration of the clinical trial should be sufficiently long, with an appropriate number of assessments, to allow the biological and clinical outcome parameters to change. All four criteria will be fulfilled by the TREFAMS-ACE programme [86,96]. Third, due to the follow-up period of 1 year, we will be able to investigate whether patients implement the newly-learned skills in daily life and whether the effect of therapy will be maintained over a longer period of time. Fourth, the baseline data of the three RCTs can be pooled, allowing several interesting cross-sectional analyses. Fifth, there is valuable support from the Dutch patient organisation, MSVN. The participation of patient organisations in health research is important when setting research agendas, during the design phase and during the study period, but it will also enhance practical relevance during later dissemination and implementation of study results [97]. Finally, a strong network of academic rehabilitation departments and MS centres will be formed, as the four study teams work together closely. This will generate high quality knowledge on the treatment of MS-related fatigue and will also promote dissemination and sharing of expertise.

Some specific issues that apply to non-pharmacological trials included in the TREFAMS-ACE programme need to be discussed [28]. Regarding the blinding procedure, everyone involved in an RCT should ideally be blinded but this is not always feasible, as is often the case in RCTs evaluating rehabilitation interventions [28,98,99]. Although patients are not blinded and the two primary outcome measures are both patient-reported, the assessment of the physical fitness parameters, the analyses of blood and salivary in the clinical chemistry laboratory and the statistical analyses of the between-group differences will all be performed by research staff blinded to the treatment allocation of the participants.

With respect to the complexity of the interventions, we decided to offer patients individual and mono-disciplinary interventions, and no multidisciplinary group intervention [100-102]. Two of the four active treatment intervention programmes, that is, CBT and ECM, have long been available and further improved in recent years [30]. The AT programme is largely based on the general principles of exercise physiology [40]. The scientific underpinnings of the valuable work of specialised MS-nurses is probably the weakest aspect.

Contamination of treatment interventions is another specific issue that might complicate the interpretation of the study results. To avoid contamination via caregivers in the same study centres, we designed three independent RCTs. Moreover, to avoid overlap between the CBT and the ECM interventions, in the developmental phase of the individual ECM protocol cognitive behavioural aspects were further specified and should now totally focus on managing energy. To prevent contamination caused by participants, all participants are requested not to start with co-interventions for fatigue during the treatment period of the study. Because of the intensity of the treatment, we expect that simultaneous interventions aimed at reducing fatigue will probably only occur in a small number of patients. Other co-interventions, for example, disease modifying drugs, are monitored throughout the study at every measurement.

Finally, the inclusion of participants may be slower than anticipated. In theory, a large number of MS patients are fatigued but it may be difficult to awaken the interest of every fatigued MS patient. To enhance participation, we arranged a number of patient-friendly measures, such as the setting-up of several study centres, travel allowance and the scheduling of appointments. In order to recruit a large group of MS patients, we enlarged our network by involving regional patient associations. In this respect, the support of and close cooperation with the Dutch patient organisation Multiple Sclerosis Vereniging Nederland (MSVN) since the design phase of the research programme has been very important.

The TREFAMS-ACE study will provide insight into the effectiveness of four mono-disciplinary rehabilitation treatment methods for MS-related fatigue in individual patients. The primary aim of these treatment methods is to reduce fatigue and to improve societal participation. Furthermore, to enhance our understanding of how these rehabilitation interventions work, a study on biological outcome measures has been added. To improve current practice, tailored and more focused rehabilitation programmes based on the most effective treatment may represent - with a clearer picture of mechanisms of action - a first step in understanding which types of patients may better respond to certain therapies. Therefore, the TREFAMS-ACE results will also be added to systematic reviews [29,30] and used to develop and update clinical practice guidelines for the treatment of MS-related fatigue [8,103].

## Trials’ status

Patient recruitment commenced in October 2011. At the time of manuscript submission 104 patients have been randomised.

## Abbreviations

AT: Aerobic training; B-IPQ: Brief Illness perception questionnaire; CBRSQ: Cognitive and behavioural responses to symptoms questionnaire; CBT: Cognitive behavioural therapy; CIRS: Cumulative illness rating scale; CIS20r: Checklist individual strength; CISS: Coping inventory for stressful situations; CNS: Central nervous system; COPM: Canadian occupational performance measure; ECM: Energy conservation management; EDSS: Expanded disability status scale; FSQ: Fatigue strategies questionnaire; FSS: Fatigue severity scale; HADS: Hospital anxiety and depression scale; HPA-axis: Hypothalamic-pituitary-adrenal axis; IECM: Individual energy conservation management; IES: Impact of event scale; ICQ: Illness cognition questionnaire; IMQ: Illness management questionnaire; IPA: Impact on participation and autonomy; MFIS: Modified fatigue impact scale; MS: Multiple sclerosis; OT: Occupational therapist; PCS: Pain catastrophising scale; PRISM: Pictorial representation of illness measure; PT: Physiotherapist; RCT: Randomised clinical trial; SAE: Serious adverse events; SES-28: Self-efficacy scale 28; SF36: Medical outcome study short form 36 items; SIP: Sickness impact profile; SSL: Social support list; TREFAMS-ACE: Treating fatigue in multiple sclerosis - aerobic training, cognitive behavioural therapy, energy conservation management; TSH: Thyroid stimulating hormone; UMCN: University medical centre Nijmegen; VO2max: Maximum oxygen consumption; Wmax: Maximum work rate.

## Competing interests

The authors declare that they have no competing interests.

## Authors’ contributions

HB and VG are the principal investigators and originated the idea for the study, developed the overall design and obtained funding for the TREFAMS-ACE research programme. MH will conduct the AT trial, LB will conduct the ECM trial and AM will conduct the biomarker study. All authors helped in finalising the manuscript. All authors read and approved the final manuscript. The TREFAMS-ACE study group advises the principal investigators and supervises the PhD students.

## References

[B1] Noseworthy JH Lucchinetti C Rodriguez M Weinshenker BG Multiple sclerosis N Engl J Med 2000 343 938 952 10.1056/NEJM200009283431307 11006371

[B2] Compston A Coles A Multiple sclerosis Lancet 2008 372 1502 1517 10.1016/S0140-6736(08)61620-7 18970977

[B3] Koch-Henriksen N Sorensen PS The changing demographic pattern of multiple sclerosis epidemiology Lancet Neurol 2010 9 520 532 10.1016/S1474-4422(10)70064-8 20398859

[B4] De Groot V Beckerman H Twisk JW Uitdehaag BM Hintzen RQ Minneboo A Lankhorst GJ Polman CH Bouter LM Vitality, perceived social support and disease activity determine the performance of social roles in recently diagnosed multiple sclerosis: a longitudinal analysis J Rehabil Med 2008 40 151 157 10.2340/16501977-0145 18509581

[B5] Kos D Kerckhofs E Nagels G D’Hooghe MB Ilsbroukx S Origin of fatigue in multiple sclerosis: review of the literature Neurorehabil Neural Repair 2008 22 91 100 17409388 10.1177/1545968306298934

[B6] Stuke K Flachenecker P Zettl UK Elias WG Freidel M Haas J Pitschnau-Michel D Schimrigk S Rieckmann P Symptomatology of MS: results from the German MS registry J Neurol 2009 256 1932 1935 10.1007/s00415-009-5257-5 19629565

[B7] DeLuca J DeLuca J Fatigue: its definition, its study, and its future Fatigue as a window to the brain 2005 Cambridge, MA: The MIT Press

[B8] Multiple Sclerosis Council for Clinical Practice Guidelines Fatigue and Multiple Sclerosis: evidence-based management strategies for fatigue in multiple sclerosis 1998 Washington, DC: Paralyzed Veterans of America

[B9] Chaudhuri A Behan PO Fatigue in neurological disorders Lancet 2004 363 978 988 10.1016/S0140-6736(04)15794-2 15043967

[B10] Krupp LB Christodoulou C Schombert H DeLuca J Multiple sclerosis and fatigue Fatigue as a window to the brain 2005 Cambridge, MA: The MIT Press

[B11] Sternberg Z Sympathetic nervous system dysfunction in multiple sclerosis, linking neurodegeneration to a reduced response to therapy Curr Pharm Des 2012 18 1635 1644 10.2174/138161212799958639 22272817

[B12] Huitinga I van der Cammen M Salm L Erkut Z van Dam A Tilders F Swaab D IL-1beta immunoreactive neurons in the human hypothalamus: reduced numbers in multiple sclerosis J Neuroimmunol 2000 107 8 20 10.1016/S0165-5728(00)00248-4 10808046

[B13] Gottschalk M Kümpfel T Flachenecker P Uhr M Trenkwalder C Holsboer F Weber F Fatigue and regulation of the hypothalamo-pituitary-adrenal axis in multiple sclerosis Arch Neurol 2005 62 277 280 10.1001/archneur.62.2.277 15710856

[B14] Induruwa I Constantinescu CS Gran B Fatigue in multiple sclerosis - a brief review J Neurol Sci 2012 323 9 15 10.1016/j.jns.2012.08.007 22935407

[B15] Amato MP Portaccio E Management options in multiple sclerosis-associated fatigue Expert Opin Pharmacother 2012 13 207 216 10.1517/14656566.2012.647767 22220738

[B16] Dodd KJ Taylor NF Shields N Prasad D McDonald E Gillon A Progressive resistance training did not improve walking but can improve muscle performance, quality of life and fatigue in adults with multiple sclerosis: a randomized controlled trial Mult Scler 2011 17 1362 1374 10.1177/1352458511409084 21677021

[B17] Dalgas U Stenager E Jakobsen J Petersen T Hansen HJ Knudsen C Overgaard K Ingemann-Hansen T Fatigue, mood and quality of life improve in MS patients after progressive resistance training Mult Scler 2010 16 480 490 10.1177/1352458509360040 20194584

[B18] Cakt BD Nacir B Genç H Saraçoğlu M Karagöz A Erdem HR Ergün U Cycling progressive resistance training for people with multiple sclerosis: a randomized controlled study Am J Phys Med Rehabil 2010 89 446 457 10.1097/PHM.0b013e3181d3e71f 20216060

[B19] Petajan JH Gappmaier E White AT Spencer MK Mino L Hicks RW Impact of aerobic training on fitness and quality of life in multiple sclerosis Ann Neurol 1996 39 432 441 10.1002/ana.410390405 8619521

[B20] Andreasen AK Stenager E Dalgas U The effect of exercise therapy on fatigue in multiple sclerosis Mult Scler 2011 17 1041 1054 10.1177/1352458511401120 21467189

[B21] Rietberg MB Brooks D Uitdehaag BM Kwakkel G Exercise therapy for multiple sclerosis Cochrane Database Syst Rev 2005 1 CD003980 10.1002/14651858.CD003980.pub2 PMC6485797 15674920

[B22] Hayes HA Gappmaier E LaStayo PC Effects of high-intensity resistance training on strength, mobility, balance, and fatigue in individuals with multiple sclerosis: a randomized controlled trial J Neurol Phys Ther 2011 35 2 10 10.1097/NPT.0b013e31820b5a9d 21475078

[B23] Van Kessel K Moss-Morris R Willoughby E Chalder T Johnson MH Robinson E A randomized controlled trial of cognitive behavior therapy for multiple sclerosis fatigue Psychosom Med 2008 70 205 213 10.1097/PSY.0b013e3181643065 18256342

[B24] Mathiowetz VG Finlayson ML Matuska KM Chen HY Luo P Randomized controlled trial of an energy conservation course for persons with multiple sclerosis Mult Scler 2005 11 592 601 10.1191/1352458505ms1198oa 16193899

[B25] Mathiowetz VG Matuska KM Finlayson ML Luo P Chen HY One-year follow-up to a randomized controlled trial of an energy conservation course for persons with multiple sclerosis Int J Rehabil Res 2007 30 305 313 10.1097/MRR.0b013e3282f14434 17975450

[B26] Finlayson M Preissner K Cho C Plow M Randomized trial of a teleconference-delivered fatigue management program for people with multiple sclerosis Mult Scler 2011 17 1130 1140 10.1177/1352458511404272 21561960

[B27] Neill J Belan I Ried K Effectiveness of non-pharmacological interventions for fatigue in adults with multiple sclerosis, rheumatoid arthritis, or systemic lupus erythematosus: a systematic review J Adv Nurs 2006 56 617 635 Erratum in: *J Adv Nurs* 2007, 57:225 10.1111/j.1365-2648.2006.04054.x 17118041

[B28] Boutron I Moher D Altman DG Schulz KF Ravaud P CONSORT Group Extending the CONSORT statement to randomized trials of nonpharmacologic treatment: explanation and elaboration Ann Intern Med 2008 148 295 309 10.7326/0003-4819-148-4-200802190-00008 18283207

[B29] Heine M Rietberg MB Van Wegen EEH Port IGL Van D Kwakkel G Exercise therapy for fatigue in multiple sclerosis Cochrane Database Syst Rev 2012 7 CD009956 10.1002/14651858.CD009956.pub2 PMC9554249 26358158

[B30] Blikman LJ Huisstede BM Kooijmans H Stam HJ Bussmann JBJ Van Meeteren J Effectiveness of energy conservation treatment in reducing fatigue in multiple sclerosis. A systematic review and meta-analysis Arch Phys Med Rehabil 2013 94 1360 1376 10.1016/j.apmr.2013.01.025 23399455

[B31] Gold SM Krüger S Ziegler KJ Krieger T Schulz KH Otte C Heesen C Endocrine and immune substrates of depressive symptoms and fatigue in multiple sclerosis patients with comorbid major depression J Neurol Neurosurg Psychiatr 2011 82 814 818 10.1136/jnnp.2010.230029 21296901

[B32] Heesen C Nawrath L Reich C Bauer N Schulz KH Gold SM Fatigue in multiple sclerosis: an example of cytokine mediated sickness behaviour? J Neurol Neurosurg Psychiatr 2006 77 34 39 10.1136/jnnp.2005.065805 16361589 PMC2117393

[B33] Flachenecker P Bihler I Cytokine mRNA expression in patients with MS and fatigue Mult Scler 2004 10 165 169 10.1191/1352458504ms991oa 15124762

[B34] Stranahan AM Lee K Mattson MP Central mechanisms of HPA axis regulation by voluntary exercise Neuromolecular Med 2008 10 118 127 10.1007/s12017-008-8027-0 18273712 PMC3010733

[B35] Castellano V Patel DI White LJ Cytokine responses to acute and chronic exercise in multiple sclerosis J Appl Physiol 2008 104 1697 1702 10.1152/japplphysiol.00954.2007 18388249

[B36] Skerrett TN Moss-Morris R Fatigue and social impairment in multiple sclerosis: the role of patients' cognitive and behavioral responses to their symptoms J Psychosom Res 2006 61 587 593 10.1016/j.jpsychores.2006.04.018 17084135

[B37] Packer T Brink N Sauriol A Managing fatigue: a six-week course for energy conservation 1995 Tucson, AZ: Therapy Skill Builders

[B38] Fischer A Heesen C Gold SM Biological outcome measurements for behavioral interventions in multiple sclerosis Ther Adv Neurol Disord 2011 4 217 229 10.1177/1756285611405252 21765872 PMC3131172

[B39] Malekzadeh A de Groot V Beckerman H van Oosten BW Blankenstein MA Teunissen C Challenges in multi-plex and mono-plex platforms for the discovery of inflammatory profiles in neurodegenerative diseases Methods 2012 56 508 513 10.1016/j.ymeth.2012.03.017 22465283

[B40] American College of Sports Medicine ACSM’s guidelines for exercise testing and prescription 2010 8 Philadelphia, PA: Lippincott Williams & Wilkins 10.1249/JSR.0b013e31829a68cf 23851406

[B41] Voet NB Bleijenberg G Padberg GW van Engelen BG Geurts AC Effect of aerobic exercise training and cognitive behavioural therapy on reduction of chronic fatigue in patients with facioscapulohumeral dystrophy: protocol of the FACTS-2-FSHD trial BMC Neurol 2010 10 56 10.1186/1471-2377-10-56 20591139 PMC2906431

[B42] Koopman FS Beelen A Gerrits KH Bleijenberg G Abma TA de Visser M Nollet F Exercise therapy and cognitive behavioural therapy to improve fatigue, daily activity performance and quality of life in postpoliomyelitis syndrome: the protocol of the FACTS-2-PPS trial BMC Neurol 2010 10 8 10.1186/1471-2377-10-8 20082714 PMC2821386

[B43] Knoop H Bleijenberg G Cognitieve gedragstherapie voor chronische vermoeidheid bij MS patiënten Nijmeegs Kenniscentrum Chronische Vermoeidheid: Behandelprotocol. Nijmegen 2011

[B44] Prins JB Bleijenberg G Bazelmans E Elving LD de Boo TM Severens JL van der Wilt GJ Spinhoven P van der Meer JW Cognitive behaviour therapy for chronic fatigue syndrome: a multicentre randomised controlled trial Lancet 2001 357 841 847 10.1016/S0140-6736(00)04198-2 11265953

[B45] Huibers MJ Beurskens AJ Van Schayck CP Bazelmans E Metsemakers JF Knottnerus JA Bleijenberg G Efficacy of cognitive-behavioural therapy by general practitioners for unexplained fatigue among employees: randomised controlled trial Br J Psychiatr 2004 184 240 246 10.1192/bjp.184.3.240 14990522

[B46] Stulemeijer M De Jong LW Fiselier TJ Hoogveld SW Bleijenberg G Cognitive behaviour therapy for adolescents with chronic fatigue syndrome: randomised controlled trial BMJ 2005 330 14 Erratum in: *BMJ* 2005, 330:820 10.1136/bmj.38301.587106.63 15585538 PMC539840

[B47] Gielissen MF Verhagen S Witjes F Bleijenberg G Effects of cognitive behavior therapy in severely fatigued disease-free cancer patients compared with patients waiting for cognitive behavior therapy: a randomized controlled trial J Clin Oncol 2006 24 4882 4887 10.1200/JCO.2006.06.8270 17050873

[B48] Goedendorp MM Peters ME Gielissen MF Witjes JA Leer JW Verhagen CA Bleijenberg G Is increasing physical activity necessary to diminish fatigue during cancer treatment? comparing cognitive behavior therapy and a brief nursing intervention with usual care in a multicenter randomized controlled trial Oncologist 2010 15 1122 1132 10.1634/theoncologist.2010-0092 20930100 PMC3227893

[B49] Jacobs HM Luttik A Touw-Otten FW De Melker RA [The sickness impact profile; results of an evaluation study of the Dutch version] Ned Tijdschr Geneeskd 1990 134 1950 1954 2234151

[B50] Van der Ploeg E Mooren TT Kleber RJ van der Velden PG Brom D Construct validation of the Dutch version of the impact of event scale Psychol Assess 2004 16 16 26 15023089 10.1037/1040-3590.16.1.16

[B51] Buchi S Sensky T Sharpe L Timberlake N Graphic representation of illness: a novel method of measuring patients’ perceptions of the impact of illness Psychother Psychosom 1998 67 222 225 10.1159/000012284 9693349

[B52] Evers AW Kraaimaat FW van Lankveld W Jongen PJ Jacobs JW Bijlsma JW Beyond unfavorable thinking: the illness cognition questionnaire for chronic diseases J Consult Clin Psychol 2001 69 1026 1036 11777106

[B53] Knoop H van Kessel K Moss-Morris R Which cognitions and behaviours mediate the positive effect of cognitive behavioural therapy on fatigue in patients with multiple sclerosis? Psychol Med 2012 42 205 213 10.1017/S0033291711000924 21672300

[B54] Dennison L Moss-Morris R Silber E Galea I Chalder T Cognitive and behavioural correlates of different domains of psychological adjustment in early-stage multiple sclerosis J Psychosom Res 2010 69 353 361 10.1016/j.jpsychores.2010.04.009 20846536

[B55] Zigmond AS Snaith RP The hospital anxiety and depression scale Acta Psychiatr Scand 1983 67 361 370 10.1111/j.1600-0447.1983.tb09716.x 6880820

[B56] Herschbach P Berg P Dankert A Duran G Engst-Hastreiter U Waadt S Keller M Ukat R Henrich G Fear of progression in chronic diseases: psychometric properties of the fear of progression questionnaire J Psychosom Res 2005 58 505 511 10.1016/j.jpsychores.2005.02.007 16125517

[B57] Kwakkenbos L van den Hoogen FH Custers J Prins J Vonk MC van Lankveld WG Becker ES van den Ende CH Validity of the fear of progression questionnaire-short form in patients with systemic sclerosis Arthritis Care Res (Hoboken) 2012 64 930 934 10.1002/acr.21618 22262505

[B58] Jacobsen PB Azzarello LM Hann DM Relation of catastrophizing to fatigue severity in women with breast cancer Canc Res Ther Contr 1999 8 155 164

[B59] Jacobsen PB Andrykowski MA Thors CL Relationship of catastrophizing to fatigue among women receiving treatment for breast cancer J Consult Clin Psychol 2004 72 355 361 15065968 10.1037/0022-006X.72.2.355 PMC2562276

[B60] Ray C Weir W Stewart D Miller P Hyde G Ways of coping with chronic fatigue syndrome: development of an illness management questionnaire Soc Sci Med 1993 37 385 391 10.1016/0277-9536(93)90268-9 8356486

[B61] Aaronson NK Muller M Cohen PD Essink-Bot ML Fekkes M Sanderman R Sprangers MA te Velde A Verrips E Translation, validation, and norming of the Dutch language version of the SF-36 health survey in community and chronic disease populations J Clin Epidemiol 1998 51 1055 1068 10.1016/S0895-4356(98)00097-3 9817123

[B62] Vercoulen JH Hommes OR Swanink CM Jongen PJ Fennis JF Galama JM van der Meer JW Bleijenberg G The measurement of fatigue in patients with multiple sclerosis. a multidimensional comparison with patients with chronic fatigue syndrome and healthy subjects Arch Neurol 1996 53 642 649 10.1001/archneur.1996.00550070080014 8929171

[B63] Van Sonderen E Sociale Steun Lijst-Interacties (SSL-I) en Sociale Steun Lijst -Discrepanties (SSL-D) 1993 Groningen: Noordelijk Centrum voor Gezondheidsvraagstukken

[B64] Sullivan MJL Bishop SR Pivik J The pain catastrophizing scale: development and validation Psychol Assess 1995 7 532

[B65] Corry M McKenna M Duggan M The role of the clinical nurse specialist in MS: a literature review Br J Nurs 2011 20 86 93 21278655 10.12968/bjon.2011.20.2.86

[B66] Beurskens AJ Bultmann U Kant I Vercoulen JH Bleijenberg G Swaen GM Fatigue among working people: validity of a questionnaire measure Occup Environ Med 2000 57 353 357 10.1136/oem.57.5.353 10769302 PMC1739950

[B67] Rietberg MB Van Wegen EE Kwakkel G Measuring fatigue in patients with multiple sclerosis: reproducibility, responsiveness and concurrent validity of three Dutch self-report questionnaires Disabil Rehabil 2010 32 1870 1876 Erratum in: *Disabil Rehabil* 2011, 33:1298 10.3109/09638281003734458 20345240

[B68] Elbers RG Rietberg MB van Wegen EE Verhoef J Kramer SF Terwee CB Kwakkel G Self-report fatigue questionnaires in multiple sclerosis, Parkinson’s disease and stroke: a systematic review of measurement properties Qual Life Res 2012 21 925 944 10.1007/s11136-011-0009-2 22012025 PMC3389599

[B69] Cardol M Beelen A van den Bos GA de Jong BA de Groot IJ de Haan RJ Responsiveness of the impact on participation and autonomy questionnaire Arch Phys Med Rehabil 2002 83 1524 1529 10.1053/apmr.2002.35099 12422319

[B70] Magasi S Post MW A comparative review of contemporary participation measures' psychometric properties and content coverage Arch Phys Med Rehabil 2010 9 S17 S28 20801275 10.1016/j.apmr.2010.07.011

[B71] Kos D Kerckhofs E Nagels G D’Hooghe BD Duquet W Duportail M Ketelaer P Assessing fatigue in multiple sclerosis: Dutch modified fatigue impact scale Acta Neurol Belg 2003 103 185 191 15008502

[B72] Krupp LB LaRocca NG Muir-Nash J Steinberg AD The fatigue severity scale. application to patients with multiple sclerosis and systemic lupus erythematosus Arch Neurol 1989 46 1121 1123 10.1001/archneur.1989.00520460115022 2803071

[B73] Van Bennekom CA Jelles F Lankhorst GJ Bouter LM The Rehabilitation activities profile: a validation study of its use as a disability index with stroke patients Arch Phys Med Rehabil 1995 76 501 507 10.1016/S0003-9993(95)80502-8 7763147

[B74] Finlayson M Preissner K Cho C Outcome moderators of a fatigue management program for people with multiple sclerosis Am J Occup Ther 2012 66 187 197 10.5014/ajot.2012.003160 22394528

[B75] Kurtzke JF Rating neurologic impairment in multiple sclerosis: an expanded disability status scale (EDSS) Neurology 1983 33 1444 1452 10.1212/WNL.33.11.1444 6685237

[B76] Linn BS Linn MW Gurel L Cumulative illness rating scale J Am Geriatr Soc 1968 16 622 626 5646906 10.1111/j.1532-5415.1968.tb02103.x

[B77] De Groot V Beckerman H Lankhorst GJ Bouter LM How to measure comorbidity. A critical review of available methods J Clin Epidemiol 2003 56 221 229 10.1016/S0895-4356(02)00585-1 12725876

[B78] Beatty WW Goodkin DE Screening for cognitive impairment in multiple sclerosis. an evaluation of the mini-mental state examination Arch Neurol 1990 47 297 301 10.1001/archneur.1990.00530030069018 2310313

[B79] Van der Ploeg HP Streppel KR van der Beek AJ van der Woude LH Vollenbroek-Hutten M van Mechelen W The physical activity scale for individuals with physical disabilities: test-retest reliability and comparison with an accelerometer J Phys Act Health 2007 4 96 100 17489011 10.1123/jpah.4.1.96

[B80] Washburn RA Zhu W McAuley E Frogley M Figoni SF The physical activity scale for individuals with physical disabilities: development and evaluation Arch Phys Med Rehabil 2002 83 193 200 10.1053/apmr.2002.27467 11833022

[B81] Fournier M De Ridder D Bensing J Optimism and adaptation to chronic disease: the role of optimism in relation to self-care options of type 1 diabetes mellitus, rheumatoid arthritis and multiple sclerosis Br J Health Psychol 2002 7 409 432 10.1348/135910702320645390 12614494

[B82] Cohan SL Jang KL Stein MB Confirmatory factor analysis of a short form of the coping inventory for stressful situations J Clin Psychol 2006 62 273 283 10.1002/jclp.20211 16299755

[B83] Schwarzer R Jerusalem M Weinman J, Wright S, Johnston M Generalized Self-Efficacy scale Measures in health psychology: A user’s portfolio Windsor: Causal and control beliefs 1995 35 37

[B84] Johns MW A new method for measuring daytime sleepiness: the Epworth sleepiness scale Sleep 1991 14 540 545 1798888 10.1093/sleep/14.6.540

[B85] Broadbent E Petrie KJ Main J Weinman J The brief illness perception questionnaire J Psychosom Res 2006 60 631 637 10.1016/j.jpsychores.2005.10.020 16731240

[B86] Teunissen CE Petzold A Bennett JL Berven FS Brundin L Comabella M Franciotta D Frederiksen JL Fleming JO Furlan R Hintzen RQ Hughes SG Johnson MH Krasulova E Kuhle J Magnone MC Rajda C Rejdak K Schmidt HK van Pesch V Waubant E Wolf C Giovannoni G Hemmer B Tumani H Deisenhammer F A consensus protocol for the standardization of cerebrospinal fluid collection and biobanking Neurology 2009 73 1914 1922 10.1212/WNL.0b013e3181c47cc2 19949037 PMC2839806

[B87] Mallik PS Finlayson M Mathiowetz V Fogg L Psychometric evaluation of the energy conservation strategies survey Clin Rehabil 2005 19 538 543 10.1191/0269215505cr789oa 16119410

[B88] Van der Werf SP Jongen PJ LA Nijeholt GJ Barkhof F Hommes OR Bleijenberg G Fatigue in multiple sclerosis: interrelations between fatigue complaints, cerebral MRI abnormalities and neurological disability J Neurol Sci 1998 160 164 170 10.1016/S0022-510X(98)00251-2 9849800

[B89] Fitzmaurice GM Laird NM Ware JH Applied Longitudinal Analysis. 2nd edition. Hoboken, NJ 2011 Wiley Series in Probability and Statistics: John Wiley & Sons

[B90] Nicholas R Rashid W Multiple sclerosis Clin Evid (Online) 2012 1202 22321967 PMC4429413

[B91] Tallner A Waschbisch A Wenny I Schwab S Hentschke C Pfeifer K Mäurer M Multiple sclerosis relapses are not associated with exercise Mult Scler 2012 18 232 235 10.1177/1352458511415143 21733890

[B92] Sim I Chan AW Gülmezoglu AM Evans T Pang T Clinical trial registration: transparency is the watchword Lancet 2006 367 1631 1633 10.1016/S0140-6736(06)68708-4 16714166

[B93] Milette K Roseman M Thombs BD Transparency of outcome reporting and trial registration of randomized controlled trials in top psychosomatic and behavioral health journals: a systematic review J Psychosom Res 2011 70 205 217 10.1016/j.jpsychores.2010.09.015 21334491

[B94] Rosti-Otajärvi EM Hämäläinen PI Neuropsychological rehabilitation for multiple sclerosis Cochrane Database Syst Rev 2011 11 CD009131 10.1002/14651858.CD009131.pub2 22071863

[B95] Khan F Turner-Stokes L Ng L Kilpatrick T Multidisciplinary rehabilitation for adults with multiple sclerosis Cochrane Database Syst Rev 2007 2 CD006036 10.1002/14651858.CD006036.pub2 PMC8992048 17443610

[B96] Golden SH Wand GS Malhotra S Kamel I Horton K Reliability of hypothalamic-pituitary-adrenal axis assessment methods for use in population-based studies Eur J Epidemiol 2011 26 511 525 10.1007/s10654-011-9585-2 21533585 PMC3697932

[B97] Abma TA Broerse JE Patient participation as dialogue: setting research agendas Health Expect 2010 13 160 173 10.1111/j.1369-7625.2009.00549.x 20536537 PMC5060528

[B98] Siemonsma PC Walker MF Practical guidelines for independent assessment in randomized controlled trials (RCTs) of rehabilitation Clin Rehabil 1997 11 273 279 10.1177/026921559701100402 9408666

[B99] Boutron I Guittet L Estellat C Moher D Hróbjartsson A Ravaud P Reporting methods of blinding in randomized trials assessing nonpharmacological treatments PLoS Med 2007 4 e61 10.1371/journal.pmed.0040061 17311468 PMC1800311

[B100] Kos D Duportail M D'hooghe M Nagels G Kerckhofs E Multidisciplinary fatigue management programme in multiple sclerosis: a randomized clinical trial Mult Scler 2007 13 996 1003 10.1177/1352458507078392 17623738

[B101] Hugos CL Copperman LF Fuller BE Yadav V Lovera J Bourdette DN Clinical trial of a formal group fatigue program in multiple sclerosis Mult Scler 2010 16 724 732 10.1177/1352458510364536 20375125

[B102] Thomas PW Thomas S Kersten P Jones R Nock A Slingsby V Green C Baker R Galvin K Hillier C Multi-centre parallel arm randomised controlled trial to assess the effectiveness and cost-effectiveness of a group-based cognitive behavioural approach to managing fatigue in people with multiple sclerosis BMC Neurol 2010 10 43 10.1186/1471-2377-10-43 20553617 PMC2905353

[B103] Anonymous Conceptrichtlijn Diagnostiek, Behandeling en Functioneren bij Multiple Sclerose 2011 Utrecht: CBO

